# Crosstalk between epithelium, myeloid and innate lymphoid cells during gut homeostasis and disease

**DOI:** 10.3389/fimmu.2022.944982

**Published:** 2022-09-16

**Authors:** Sonia Ghilas, Ryan O’Keefe, Lisa Anna Mielke, Dinesh Raghu, Michael Buchert, Matthias Ernst

**Affiliations:** ^1^Mucosal Immunity Laboratory, Olivia Newton-John Cancer Research Institute, and La Trobe University - School of Cancer Medicine, Heidelberg, VIC, Australia; ^2^Cancer and Inflammation Program, Olivia Newton-John Cancer Research Institute, and La Trobe University - School of Cancer Medicine, Heidelberg, VIC, Australia

**Keywords:** intestinal epithelium, macrophages (MΦ), dendritic cells (DC), innate lymphoid cells (ILC), homeostasis, inflammation, cancer

## Abstract

The gut epithelium not only provides a physical barrier to separate a noxious outside from a sterile inside but also allows for highly regulated interactions between bacteria and their products, and components of the immune system. Homeostatic maintenance of an intact epithelial barrier is paramount to health, requiring an intricately regulated and highly adaptive response of various cells of the immune system. Prolonged homeostatic imbalance can result in chronic inflammation, tumorigenesis and inefficient antitumor immune control. Here we provide an update on the role of innate lymphoid cells, macrophages and dendritic cells, which collectively play a critical role in epithelial barrier maintenance and provide an important linkage between the classical innate and adaptive arm of the immune system. These interactions modify the capacity of the gut epithelium to undergo continuous renewal, safeguard against tumor formation and provide feedback to the gut microbiome, which acts as a seminal contributor to cellular homeostasis of the gut.

## Introduction

In mammals, the intestine forms a vital organ for the processing of food, while also functioning as a barrier that protects the host from ingested pathogens and external noxious stimuli. The intestinal environment is highly sophisticated and controlled, harboring commensal microorganisms such as bacteria, viruses, and fungi, establishing a mutualistic symbiotic relationship with the host. Through tightly controlled structural organization, the intestinal epithelium, enteric neurons, gut-resident immune cells and other minor cell populations collectively cooperate with the microbiota to achieve a harmonious environment.

**Figure 1:**
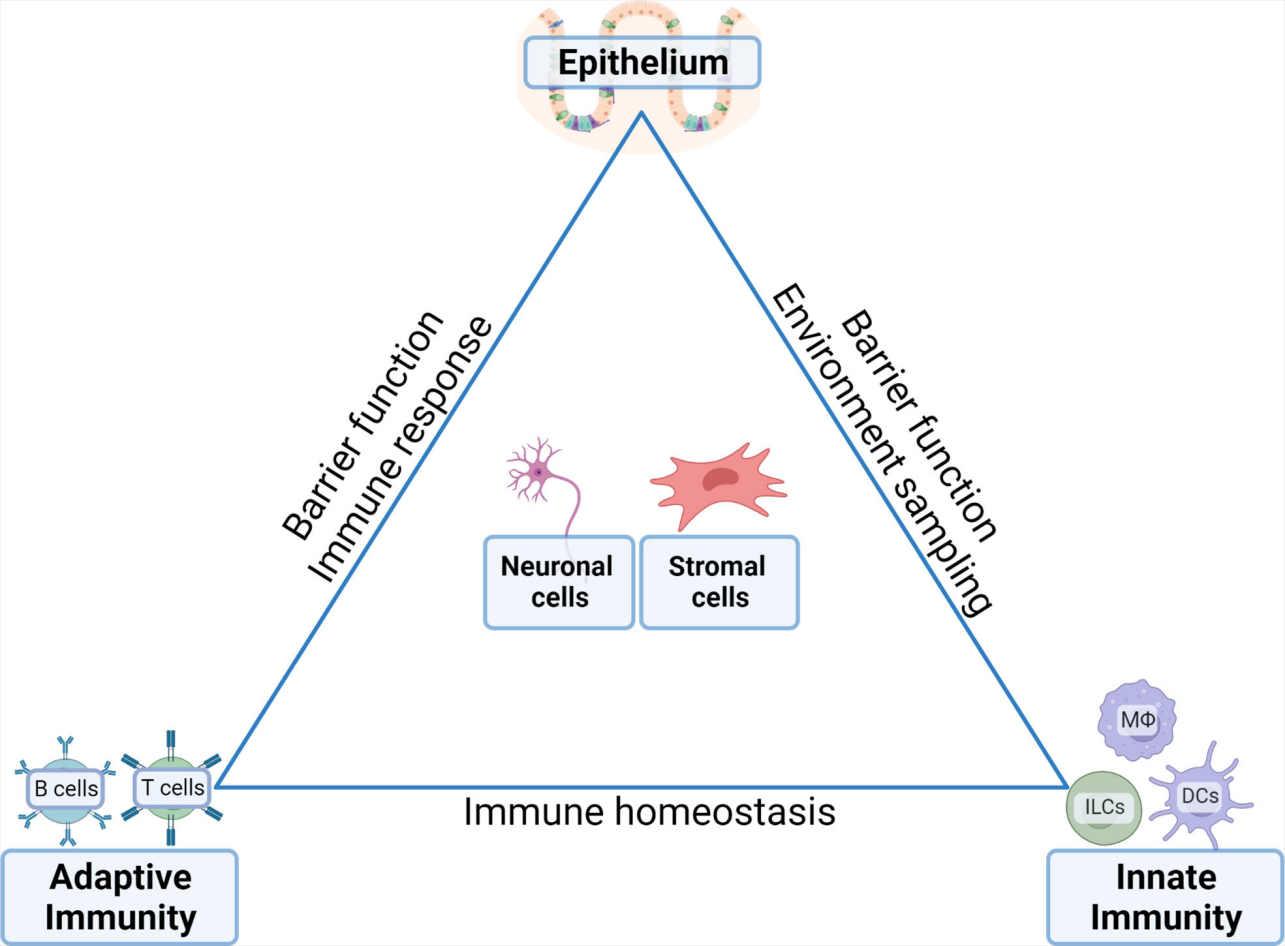
Communication between distinct cell compartments ensures tissue homeostasis in the gut. The complex function and need for rapid adaptability to external stimuli (i.e. food, water, bacterial load and products, enteric signals) requires carefully balanced and controlled equilibria between the epithelia, the innate immune compartments, the adaptive immune compartments and their reciprocal interactions with enteric neuronal network and stromal cells. Inflammatory conditions arise in situations where the balances are skewed by epithelial signals that stimulate the immune compartments and a disbalance between the innate and adaptive compartments. In neoplastic situations, the transformed epithelium feeds of the cytokine-rich environment established by the immune compartments including the establishment of an immune suppressive bias towards anti-tumor immunity conferred by tumor-eradicating effectors cells.

The intestinal epithelium is a tight monolayer that separates the host from the lumen, which contains digestive enzymes, food, and microbes from the external environment. Stem cells in the crypt constantly replenish stressed or damaged epithelial cells to maintain this barrier (1). The intestinal epithelium encompasses specialized cells with important functions in maintaining homeostasis. Among them are goblet cells, which secrete mucus forming a protective layer in the lumen, Paneth cells which secrete antimicrobial agents working together to prevent the entry of the luminal microbes into the host, and tuft cells which act as sentinel cells that scan the lumen with their long brush-like microvilli projections (2). In addition, the epithelial layer contains tissue-resident lymphocytes, known as intraepithelial lymphocytes (IELs) that primarily comprise of T cell receptor (TCR) αβ T cells, TCR γδ T cells, and smaller populations of type I innate lymphoid cells (ILC1s). Cellular homeostasis in the gut is achieved through the impeccable functioning of these tissue-resident lymphoid cells that reside or closely interact with the epithelium (**Figure 1**).

Localized underneath the epithelium is the lamina propria, a large layer of loose connective tissue that forms part of the intestinal mucosa. The lamina propria contains most of the immune components of the gut, with tiny lymphoid aggregates named cryptopatches, isolated lymphoid follicles, and bigger clusters of organized lymphoid follicles known as Peyer’s patches. Innate and adaptive immune cells accumulate in these lymphoid aggregates, where they can directly interact with the epithelium (3). Indeed, IELs, macrophages and dendritic cells are essential for maintaining epithelial barrier integrity and gut homeostasis. Moreover, IELs play critical roles in sensing epithelial cell stress (4), control bacterial composition in the lumen (5), detect invading pathogens (6) and develop tolerance towards dietary or innocuous antigens (2, 7). Complex interdependencies between the epithelial and gut-resident immune cells are the key to maintain homeostasis, responding to allergens as well as preventing infection, or the development of inflammatory bowel disease (IBD), such as Crohn’s disease (CD) or ulcerative colitis (UC), and cancer.

Studies aiming to develop therapeutics that target either the intestinal epithelium or accompanying immune cells and aimed to detect or eliminate neoplastic epithelium require a clear understanding of the intricate interaction between epithelium and immune cells. The role of IELs in establishing and maintaining intestinal tolerance and immunity has been widely reviewed (8), therefore, we will focus here on the functions of innate lymphoid cells, myeloid cells and cellular crosstalk between these populations. We will review current knowledge of how these two immune cell types interact with the intestinal epithelium to maintain homeostasis, and how those interactions are affected during disease development and progression.

## Homeostasis of the intestinal mucosa

### Myeloid cells in the intestinal mucosa

Under homeostatic conditions, myeloid cells are one of the most abundant immune cell types in the gastrointestinal tract (9). They comprise a very heterogeneous population, encompassing among others, granulocytes, neutrophils, monocytes, myeloid-derived suppressor cells, macrophages, and dendritic cells. In this review, we mainly focus on macrophages (MФs) and dendritic cells (DCs), which are found in the mucosa throughout the gastrointestinal tract and primarily reside in the lamina propria, immediately adjacent to the epithelium (10). DCs, which develop from bone marrow progenitors, accumulate in defined gut structures such as Peyer’s patches, isolated lymphoid follicles, and gut-associated lymphoid tissues. Like DCs, MФs are continuously replenished from bone marrow-derived progenitors. In addition, the gut also contains self-maintaining embryonic-derived macrophages in the close vicinity of enteric neurons, blood vessels, Peyer’s patches, and epithelial Paneth cells (11). Owing to their strategic position and various functions, myeloid cells help maintain a balance between homeostasis and inflammation in the gut.

MФs are highly plastic cells with a spectrum of endotypes reaching from proinflammatory, immune-permissive and tumor-restricting M1-/conventionally activated MФs to anti-inflammatory, immune-restricting, and tumor permissive M2-/alternative activated MФs. It is now clear that MФs endotypes are largely under the control of environmental cues, where cytokines like interferon (IFN)-γ or granulocyte-macrophage colony-stimulating factor (GM-CSF) and bacterial products, including LPS, enhance the development of conventionally polarized MФs that express major histocompatibility complex class II (MHC-II) and CD80. These M1-like MФs produce T helper (T_H_) 1 responses-inducing cytokines, including interleukin (IL)-1β, IL-12, IL-18, IL-23, tumor necrosis factor alpha (TNF-α) and inducible nitric oxide synthase (iNOS). Therefore, conventionally polarized MФs are associated with host defense, high microbicidal activity and pro-inflammatory cytokine production; accordingly, M1-like MФs can become key mediators of autoimmune diseases when aberrantly activated. By contrast, environments rich in IL-4, IL-13, CSF-1 or transforming growth factor beta (TGF-β) result in alternative activated MФs that express CD206, CD163 and Arginase 1, and produce T helper (T_H_)2-cytokines (IL-6, IL-10 or VEGF) alongside various monocyte attracting CC chemokine ligands (CCL) chemokines [reviewed in (12)]. In the murine and human systems, these alternative activated MФs promote debris scavenging, tissue repair and wound healing, besides promoting fibrosis (13).

The phenotype and transcriptional profile of alternative activated, M2-like MФs differs considerably depending on the factors under which polarization occurs. Integrated phenotypic analysis through transcriptomic, protein profiling and metabolomic characterization re-emphasizes the somewhat fluid continuum between the various endotypes and reflecting variability between the interaction of MФs and their environments (14). Nevertheless, M2-like MФs are traditionally segregated into CD206 expressing M2a MФs that arise in response to the Th2 cytokines IL-4 or IL-13, CD163-expressing M2c MФs arising in response to IL-10 and glucocorticoids, and angiogenic (VEGF-producing) M2d MФs which are induced by IL-6 and Toll-like receptors (TLR) activation. An additional major subset comprises CD86-expressing regulatory (M2b) MФs that are activated in response to either TLRs, IL-1 or high-density immune complexes, adenosine, prostaglandin, and other mediators (15). The latter stimuli lead to activation of multiple transcription factors such as nuclear factor kappa-light-chain-enhancer of activated B cells (NF-κB), mitogen-activated protein kinases (MAPK) and interferon regulatory factor 3 (IRF3), as well as phosphoinositide 3-kinases (PI3K) signaling (16). Regulatory MФs confer potent anti-inflammatory activities, as they produce high levels of IL-10 at the expense of pro-inflammatory cytokines (17). Importantly, and owing to the transient state of MФs, they are best characterized by their functional state of activation (i.e. their cytokines, see above).

While in most tissues under homeostatic conditions, MФs are primarily derived from bone-marrow progenitors, tissue-resident MФs in the adult intestine are continuously replaced from circulating Ly6C^High^ monocytes (18) or from circulating CD14^+^ monocytes in human and mice (19), where they replace the yolk sac-derived MФs in the embryo. Thus, MФs within the intestine comprise a heterogeneous mixture of cells with a self-maintaining population, arising from embryonic precursors and adult bone-marrow-derived monocytes that persist throughout adulthood (11). Interestingly, depletion of the self-maintaining populations alters the submucosal vasculature and triggers degeneration of enteric neurons resulting in altered muscle contractility and neuron-dependent secretion of anions in the lamina propria (11).

**Figure 2:**
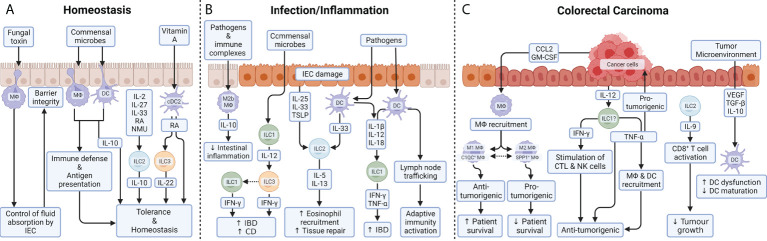
Myeloid and innate lymphoid cell function in the colon. **(A)** During homeostasis, balloon-like protrusions formed by LP-MФs sense and limit absorption of fungal toxins by IECs and thus control epithelial integrity. As a large proportion of the luminal microbes are commensals, MФs and DCs must be tolerogenic to prevent inflammation in homeostatic conditions. Their production of IL-10 ensures low responsiveness to stimulation through TLR and balances T cell activity during homeostasis. Under steady-state conditions, a small proportion of intestinal ILC2s also express the anti-inflammatory cytokine IL-10 upon exposure to a variety of exogenous stimuli including IL-2, IL-4, IL-27, IL-10 or neuromedin U (NMU). While the role of ILC2-derived IL-10 remains unclear, it could help to maintain the intestinal mucosa in an anti-inflammatory state. DCs are able to metabolize vitamin A into RA in the intestine. ILC3 can respond to RA, thanks to the transcription factor retinoic acid receptor (RAR) which stimulates IL-22 production under homeostasis as well as during colitis. **(B)** During inflammatory diseases, an imbalance in the composition of ILC subsets is commonly observed and is thought to contribute to pathogenesis. IL-12 signaling (released by ILC1) gives ILC3 cells the ability to produce IFN-γ. Phenotypic conversion of ILC3 to IFN-γ producing ILC1 is prominent in patients with CD, supporting the pathologic role for IFN-γ secreted from either ILC1 or ILC3. Tissue-resident ILC2s respond to tissue damage and a variety of pathogen-associated danger signals through the expression of receptors for alarmins, such as IL-25R, IL-33R, and thymic stromal lymphopoietin (TSLP) receptor. IL-5 and IL-13 mediate the recruitment of eosinophils and promote tissue repair. After exposure to microbial metabolites, DCs produce IL-12, IL-18 and IL-1β, which are the dominant cytokines required for the induction of IFN-γ and TNF-α by ILC1 during gut inflammation. In response to TLR ligands and immune complexes, a subset of MФs, called regulatory M2b MФs, produce high levels of IL-10 which help reducing intestinal integrity during inflammation. **(C)** In colorectal carcinomas, cancer cells produce GM-CSF and CCL2 which trigger the recruitment of MФs into the tumor microenvironment. In tumor stroma, a high M1/M2 density ratio was associated with better cancer-specific survival, while preclinical models suggest that genetic or pharmacologic suppression of the M1 to M2 endotype transition reduces colon cancer. In parallel, two populations of MФs, that do not strictly correspond to the M1 or M2 macrophages, have been identified in CRC patients: C1QC^+^ MФs and SPP1^+^ MФs. Complement C1q C chain positive MФs (C1QC^+^ MФs are enriched for complement activation and antigen processing/presentation pathways, indicating their role in anti-tumor responses; whereas secreted phosphoprotein positive MФs 1 SPP1^+^ MФs express genes involved in tumor angiogenesis and tumor vasculature, suggesting that they play a pro-tumorigenic and pro-metastatic role in CRC. While TNF-α is widely reported to be pro-tumorigenic, the release of IFN-γ and TNF-α by ILCs are believed to be anti-tumorigenic: IFN-γ through stimulation of cytotoxic T cells (CTL) and NK cells and TNF-α through direct induction of apoptosis in tumor cells and tumor vasculature and indirectly through mobilization of MФs and DCs. While ILC1s promote chronic intestinal inflammation *via* the production of IFN-γ and TNF-α, those same cells are a potential candidates ()? to therapeutically induce IFN-γ and TNF-α for tumor suppression. ILC2s are abundant in colon cancer tissue and are the dominant source of IL-9 which can activate CD8^+^ T cells to inhibit tumor growth. Colon cancer antigens can induce DCs recruitment, maturation, and cytokine release in order to generate effective T_H_1-type immune responses. Immunosuppressive signals released by tumor cells or immunomodulatory cells, such as TGF-β, VEGF or IL-10 induce DC dysfunctionality, by inhibiting their production of pro-inflammatory cytokines, and/or prevent DC maturation.

The lamina propria macrophages (LP-MФs)are classically defined as CD64^+^ CD11c^+^ MHC-II^High^ (and in mice also CX3CR1^High^) sessile cells with pseudopods that form transepithelial dendrites (TEDs) (20). These structures cross the epithelial barrier to endow LP-MФs with the capacity to sample the intestinal lumen and capture potential antigens. Recently, balloon-like protrusions, formed by LP-MФs,that sense and limit absorption of fungal toxins by intestinal epithelial cells (IECs), have been identified into the colonic epithelium (21). Unlike TEDs, these protrusions do not reach the lumen of the colon, but rather internalize membranes from IECs and examine the presence of fungal products. If fungal metabolites are detected, IECs stop fluid absorption to prevent their poisoning and associated apoptosis (21). Therefore, thanks to their protrusions, distal colon MФs are able to maintain local homeostasis by helping the epithelium to maintain its integrity **(** **Figure 2A**). Whether alteration of this mechanism lead to pathologies has not been addressed yet. LP-MФs have been shown to have a more inflammatory endotype than their counterparts, and are buried further away from the epithelium in the muscularis mucosae (22). Nevertheless, as a large proportion of the luminal microbes are commensals, LP-MФs must be tolerogenic to prevent inflammation in homeostatic conditions and therefore express only low levels of IL-1, IL-6, TNF-α and other inflammatory mediators. Instead, their production of IL-10, and response to it, ensures low responsiveness to stimulation through TLRs (23, 24) and balances T cell activity during homeostasis (**Figure 2A**). Indeed, it has been observed that the uptake of apoptotic IECs induced a transcriptional program associated with immunosuppression, including down-regulation of TLR2, likely as a mechanism to prevent unwanted inflammatory or autoimmune responses (25). The critical role for IL-10 and associated signaling is highlighted by the spontaneous enterocolitis that develops in mice which either lack expression of the IL-10 receptor alpha chain in macrophages (26), or myeloid cell-specific expression of the IL-10 signaling-associated transcription factor Stat3 (27).

As the name implies, the long-lived muscularis macrophages (M-MФs) are embedded with the muscularis mucosae. M-MФs are MHC-II^+^, CD163^+^, CX3CR1^+^ cells that do not migrate under homeostatic conditions, and are seeded both as bone marrow- and yolk sac-derived c-Myb^+^ cells (11). M-MФs not only phagocytose dying neurons and neuronal debris in the small and large intestine (28), but also play an important role in regulating intestinal motility (29) through their bi-directional interactions with glia and neurons. For instance, M-MФs produced bone morphogenic protein type 2 that contributes to neuronal functions, while enteric neurons constitutively produce CSF-1, which is needed for maintenance and survival of M-MФs (30). Unlike LP-MФs,little is known about the function of M-MФs. Nevertheless, transcriptomic analyses have shown that M-MФs are skewed towards M2 endotypes under homeostatic conditions. M-MФs preferentially express genes associated with tissue-protection and wound-healing, including as *Retnla*, *Cd163* and *Il-10* (22), suggesting a role in homeostatic tissue repair. Indeed, the tissue-protective functions of M-MФs is emphasized by their maintenance of M2-like endotypes even when homeostasis is disrupted by bacterial pathogens (22).

Akin to MФs, DCs can either develop from bone-marrow progenitor cells during homeostasis or from monocytes during inflammation. These precursors give rise to two populations of cells with different features and functions: homeostatic DCs or monocyte-derived inflammatory DCs, respectively. Homeostatic DCs are highly heterogeneous and comprise PDCA-2^+^ plasmacytoid DCs (pDC) and conventional or classical DCs (cDC). The latter can be further segregated into chemokine (C motif) receptor^+^ (XCR1), or type 1 cDCs (cDC1) and signal-regulatory protein alpha^+^ (SIPRα), or type 2 cDCs (cDC2). Intestinal cDC2 are themselves a heterogeneous population, whereby cDC2 as in the LP can be either CD103^+^ or CD103^-^. Each of these subpopulations harbors its unique functional, phenotypical, and transcriptional characteristics. While cDC1 are well known for their excellent capacity to cross-present antigens to cytotoxic lymphocytes, cDC2 and pDCs are known to polarize CD4^+^ T cell responses and to induce anti-viral responses through type I IFN production. The main function of DCs is to link innate and adaptive immunity by sensing and capturing pathogens and triggering adaptive responses specific to the pathogens detected. Like LP-MФs, intestinal DCs take up soluble food antigens through TEDs (20), but also through epithelial M-cells, which are located in the follicle-associated epithelium of Peyer’s patches (31). Moreover, CX3CR1^High^ LP-MФs can transfer antigens to DCs *via* a Connexin 43-dependent mechanism at gap junctions (32, 33). It also should be noted that at least in chronic inflammatory conditions, CX3CR1^High^ LP-MФs are endowed with migratory capacity and can invade mesenteric lymph nodes in a similar manner to migrating DCs (24). While there is general consensus that DCs from a healthy intestinal tract are tolerogenic upon sensing of commensal bacterial components, the mechanisms by which this is achieved are not fully understood. cDC1 have been shown to be crucial for intestinal homeostasis, notably owing to their expression of the chemokine receptor XCR1. Indeed, mice with XCR1-deficient cDC1 lack intraepithelial and LP T cell populations and are remarkably more susceptible to chemically-induced colitis (34). CD103^+^ cDC2 are believed to be important in establishing oral tolerance in part due to their ability to produce retinoic acid (RA) necessary for the development of Foxp3^+^ regulatory T (Treg) cells (35, 36). Moreover, mammalian target of rapamycin mTOR protein kinase has been shown to regulate intestinal homeostasis by promoting IL-10 production in cDC2. Indeed, the lack of mTOR signalling specifically in DCs resulted in the suppression of IL-10 production by cDC2 and a higher susceptibility to dextran sodium sulfate (DSS)-induced colitis (37).

### Innate lymphoid cells in the intestinal mucosa

Tissue-resident helper ILC have emerged as pivotal sentinels of gastro-intestinal tissue homeostasis, occupying strategic defensive positions along the various intestine and gut-associated lymphoid tissues. ILCs can raise antigen-independent immune responses, making them ideal gatekeepers for barrier tissues. The quality of ILC-driven immune responses at a mucosal site is determined by the level of cytokine activation, apoptosis, and the proliferation and differentiation of any given ILC subtype, as well as tissue-specific migration and accumulation of peripheral ILCs. Among the ILC populations, three main subtypes exist, referred to as ILC1, ILC2 and ILC3, which are distinguished by the expression of transcription factors required for their differentiation/maintenance, and their specific expression profiles of effector cytokine. The highest frequency of ILCs is found in the gut (38), where under homeostatic conditions, the dominant subtype is NKp44^+^ ILC3, with only low numbers of ILC2s (38–41). The frequency of ILC populations drastically changes in IBD patients (41), with decreased numbers of NKp44^+^ ILC3 and increased number of both ILC1s and ILC2s. CD patients have increased ILC1, as well as IL-17 and IL-22 producing ILC3 (41–43), while patients with UC have increased numbers of ILC2 (41). Together, this indicates subsets-specific, location-specific and disease-specific ILC immune responses, most likely refined by factors in the local epithelium.

Under homeostatic conditions, two major ILC1 subsets reside in the intraepithelial compartment and in the LP of the mammalian intestine (44, 45). Most intraepithelial ILC1 cells express CD49a and CD69 markers for tissue retention and stain positively for the transcription factors Eomes and T-bet (46, 47). Induced loss of T-bet expression using RosaCreERT2 mice results in depletion of ILC1, but not of ILC2 or ILC3. In contrast, lamina propria ILC1 cells express low levels of Eomes in favor of marked expression of IL7R and IL15R alpha chains (48).

Recent studies suggest potential continuity across specific ILC subtypes, including trans-differentiation between specific subtypes. This has been observed in murine colorectal cancer (CRC) tumor models, where IL-22 producing ILC3 trans-differentiated into IL-10 producing regulatory ILCs (ILCregs), thereby promoting CRC. This conversion is mediated by TGFβ, as inhibition of TGFβ disrupts ILC3 conversion and curbed tumor growth (49). Additionally, ILC3 to ILC1 plasticity has been reported in mice, where intestinal ILC3s deficient for the key regulator genes BCL6 and cMAF, trans-differentiated into an INF-γ-producing ILC1-like phenotypes (49). Finally, a shift from ILC2 to INF-γ-producing ILC1 has also been observed during inflammation in mice and humans caused by chronic obstructive pulmonary disease, viral or bacterial infection (40, 50). Interestingly, ILC3 to /ILC1 and ILC2 to /ILC1 conversions are both reversible, with increased IL-23, IL-2, IL-1β and RA resulting in the reversal of ILC3 to ILC1 (51). While the reversal process from ILC2 to ILC1 is not fully understood, ILC2s expressing receptors for IL-1β, IL-12, IL-18, and IL-33 are more likely to undergo trans-differentiation, while ILC2s exposed to eosinophil-produced IL-4 are less likely to do so (40, 50).

Gut resident ILC2s are defined by their expression of the Gata3 and Rorα transcription factors, as well as the secretion of the type 2 effector cytokines IL-4, IL-5, IL-9 and IL-13. Tissue-resident ILC2s respond to tissue damage and a variety of pathogen-associated signals though the expression of receptors for alarmins (IL-25, IL-33 and thymic stromal lymphopoietin (TSLP)), prostaglandins and interferons. The development of ILC2 populations in the murine gut is shaped by local immune populations and microbiota in an IL-25 and IL-33 dependent manner. While IL-25 stimulates an inflammatory subtype of ILC2s, called inflammatory ILC2 (iILC2), IL-33 shapes the natural ILC2 (nILC2) population which predominantly expresses the IL33 receptor (ST2) (52–55).

Under homeostatic conditions, a small proportion of murine intestinal ILC2s express the anti-inflammatory cytokine IL-10 after exposure to a variety of exogenous stimuli, including IL-2, IL-4, IL-10, IL-27 and the neuropeptide neuromedin U (NMU) (56) (**Figure 2A**). While the physiologic relevance of ILC2-derived IL-10 remains unclear, it is tempting to speculate that it helps to maintain the intestinal mucosa in an anti-inflammatory state (57). Certain luminal contents, such as succinate, activate murine intestinal ILC2s indirectly via tuft cells, which are the dominant epithelial source of the alarmin IL-25 (58). Intestinal infection with certain parasites (e.g., helminths) triggers ILC2 activation in a similar mechanistic fashion to the indirect activation of ILC2 by succinate. In the latter situation, parasite-sensing tuft cells release IL-25 and leukotrienes C4 (LTC_4_) to activate ILC2s which express the IL-25 and LTC_4_ receptors (59). In turn, ILC2-derived IL-13 skews the differentiation of murine intestinal stem cells towards goblet and tuft cells at the expense of absorptive enterocytes. In an effort to rebalance the absorptive capacity of the intestine, while also maintaining full immunity against the parasite and protection against re-infection, crypt fission leads to a lengthening of the intestine within weeks (59). Of note, in the colon where both succinate and helminth infections fail to induce tuft and goblet cell hyperplasia, an alternative IL-33/IL-13 circuit exists that expands tuft and goblet cells. In this case, IL-33 released from colonocytes activates stromal cells which secrete IL-13 leading to an increase in tuft and goblet cells.

Stimulation of ILC2s with IL-33 does not only induce ILC2 proliferation, but also accelerate their migration. In mice, intestinal inflammation leads to the accumulation of nILC2s in the lung *via* IL-33/CXCL6 mediated migration of nILC2s from the intestine to the lung. Interestingly, nILC2s upregulate the CXCR6 receptor during inflammation, while iILC2 accumulation in the intestine appears to be mediated by IL-25 and CCL25 (60). Another report has shown that the migratory potential and cell fitness of murine iILC2 is regulated by the AP-1 superfamily protein (basic leucine zipper ATF-like transcription factor (BATF) (61). Indeed, increased migratory potential of activated ILC2s was also observed during helminth infections and this was accompanied by prolonged interactions with T cells in the inflamed mucosa, indicating that contact-based T cell activation is mediated by activated ILC2s (62).

ILC3 are the major ILC subtype in the mammalian gut, to where they are seeded as immature precursor cells which mature into two major populations that differ developmentally, phenotypically and functionally. Lymphoid tissue inducer cells (LTi)-like ILC3 cells which express the surface receptor CCR6, and NKp46^+^ ILC3s (63). The maturation of ILC3s is regulated by diverse factors, including RA, diet-derived polyphenols and the microbiota (64). In mice, ILC3s are critical for the establishment of cryptopatches and intestinal lymphoid follicles near the epithelial layer, and assist in maintaining the integrity of the intestinal barrier and tissue homeostasis by regulating microbiota content (65, 66). ILC3 sense a wide variety of environmental signals originating from the diet, microbiota or surrounding cells; in response ILC3 release cytokines including IL-17A/F, IL-22, GM-CSF and IL-2. IL-22 and IL-17 expression in ILC3s follow the patterns of circadian oscillations. Deletion of the circadian clock regulator gene *REV-ERBa* in murine ILC3s, leads to their impaired development of the NKp46^+^ but not NKp46^-^ (LTi-like ILC3) population, thereby reducing IL-22 expression, and increasing IL-17 expression (67). Several genes necessary for the maintenance of the circadian clock, such as brain and muscle ARNT-Like 1 (*BMAL1*), are highly expressed in ILC3s. These genes, along with many ILC3 effector genes are expressed in a diurnal oscillatory fashion. Deletion of *BMAL1* resulted in reduced numbers of ILC3s in the murine gut, as well as the induction of certain hyperactivated features, which were partially restored after the depletion of the gut microbiota (68). Similarly, ablation of the circadian regulator Arntl in murine ILC3s led to disruptions in gut homeostasis, lipid metabolisms and increased susceptibility to colitis (69). Notably, IBD patients show deregulated expression of circadian-related genes (68). Overall, this underlines that correct circadian regulation of ILC3 activities is required for intestinal homeostasis, with their deregulation leading to the development of gut disease.

## Cellular crosstalk in the intestinal mucosa

### Crosstalk between myeloid cells and innate lymphoid cells

LP-MФimmune responses such as IL-6, IL-23, IL-1rn ands are critically involved in controlling the maintenance and activation of intestinal ILCs and T cells, including Treg cells. LM-MФs derived IL-10 not only affects CD4^+^ T cell responses and differentiation of Foxp3^+^ Treg cells (70, 71), but LM-MФs can also induce IL-10 production by neighbouring T-cells (70). While the production of IL-1β by a subset of resident LP-MФs is important for the development of T_H_17 cells under homeostatic conditions (71), CX3CR1^+^ mononuclear phagocytes can also prime T cells towards T_H_17 cell differentiation. DCs and LP-MФs have also been found to be regulated through the GM-CSF, promoting DCs and LP-MФs driven protection against environmental pathogens, with increased GM-CSF being identified in humans and mice undergoing an inflammatory response (72–76). In the GI tract of mice, a subset of ILC3s expressing RORγt^+^ have been identified as the primary source of GM-CSF during inflammation. In response to increased IL-1β produced by microbe-sensing LP-MФs through the TLR-adapter protein Myd88, DC and LP-MФs release IL-10 and RA which in turn trigger the conversion of naïve T cells into Tregs and contribute to murine intestinal homeostasis (77). On the other hand, LP-MФs production of IL-23 and IL-1β is kept under control by LAG3^+^ Treg cells (78), suggesting a feedback loop, in which pro-inflammatory LP-MФs induce Treg cells to contribute to a tolerogenic milieu.

Myeloid derived cells such as DCs and MФs in the mammalian gut are key regulators of ILCs. After sensing bacterial metabolites *via* TLRs, myeloid DCs secrete pro-inflammatory cytokines such as IL-1α, IL-1β, IL-12, IL-18, IL-23, IL-33 and the TNF family member TL1A. IL-12 activates ILC1s to secrete IFN-γ and TNF-α with IL-12-responsiveness being especially acute in intraepithelial NKp44^+^ CD103^+^ ILC1 (46). In mice, IL-1β and IL-23 induce the expression of MHCC -class II molecules on ILC3. Meanwhile, TL1A binds death receptor 3 (DR3) expressed on ILC3 and induces the expression of the co-stimulatory molecule CD40L which then facilitates differentiation of T_H_1 and T_H_17 cells (but not Tregs) and expression of IL-2 receptor subunit CD25 on TL1A stimulated ILC3. This presumably primes ILC3s to become responsive to the mitogenic IL-2 signal (79, 80).

ILC3s in cryptopatches (CPs) and isolated lymphoid follicles (ILF) directly interact with cDCs *via* expression of lymphotoxin-αβ (LTα1β2). cDC associated with CPs and ILFs are transcriptionally distinct from other tissue resident cDCs. In particular, they express genes associated with regulation of immune responses such as IL-6, IL-23, IL-1rn and the IL-22 binding protein (IL-22BP), which blocks IL-22 activity. The latter expression profile is dominant in ILF-associated cDCs. ILF-cDCs are absent in ILC3-deficient mice and mice unable to express IL-22BP from ILF-cDCs have significant reduction in the expression of genes required for lipid uptake in intestinal epithelial cells leading to whole body alterations in lipid homeostasis (81). This result is in agreement to data showing that the vasoactive intestinal peptide suppressed IL-22 expression in ILC3 and associated increase in lipid uptake after food intake (82, 83).

cDC2 cells metabolize vitamin A into RA in the intestine. ILC3 express the RA receptor and RA stimulates IL-22 production during homeostasis as well as during colitis in mice (84). All-trans RA also induces IL-22 in ILC3 *via* the HIC1 transcription factor and this protects the murine gut from invasion by pathogens (85). Conversely, lack of RA leads to a loss of proliferating ILC3s and LTi cells resulting in reduced lymphoid organ development and the induction of fewer secondary lymphoid organs in mice (86, 87) (**Figure 2A**).

Transcriptional profiles of MФs isolated from colons of DSS-challenged mice suggested regulation by GM-CSF. ILC3s are the principal source of GM-CSF in the colon after DSS treatment with lower contribution from γδ T cells and ILC2s. GM-CSF augments MФ activation towards inflammatory signals (i.e. expressing IL-1β, IL-12/23) during colitis in both humans and mice. Anti-GM-CSF treatment leads to defective monocyte maturation, i.e. accumulation of immature monocytes and improved colitis. MФs unable to respond to GM-CSF led to reduced IL-22, but not IL-17A production, by ILC3. GM-CSF promotes M1 polarization of MФs while suppressing the M2 endotype of MФ (88).

### Crosstalk between myeloid cells and intestinal epithelial cells

Through their capacity to sample the “outside”, myeloid cells are important regulators for IECs to retain their barrier functions. For instance, myeloid cell-produced IL-10 regulates the microbiota-dependent increase in pro-inflammatory mediators such as IL-23, as revealed by the analysis of IL-10-deficient mice. In turn, IL-23 induces production of IL-22 and IL-17 in T_H_17 and ILC3 cells (26). Subsequently, IL-22-activated Stat3 in IECs elicits transcription of survival and proliferative gene signatures, as well as the expression of antimicrobial peptides which trigger a subsequent recruitment of neutrophils. Under regenerative conditions, for instance in response to induction of acute colitis following administration of the luminal irritant DSS, or high level of γ-irradiation, the Stat3 response in IECs is critical to match the epithelial repair to the extent of inflammation arising from the break-down in epithelial barrier function. We have previously termed this as a rheostat function, whereby myeloid and stromal derived activators of Stat3 signaling in IECs (i.e. IL-6, IL-11) provide a temporary boost to the homeostatic proliferation activity within the intestinal epithelial stem cell compartment that is controlled by canonical Wnt-signaling (89, 90). Besides Stat3 activity in IECs providing a central signaling node to ensure myeloid cell communication with IECs, other soluble mediators for the stimulation of proliferation of epithelial progenitors in intestinal crypts include the lipid mediator prostaglandin E_2_, IL-1 and others (91, 92). More recent studies also suggest that the myeloid cell/IEC crosstalk can occur through the production of ribonuclease angiogenin by LP-MФs, which supports IECs growth and survival (93). MФs directly affect homeostatic renewal of IECs, as MФs ablation following CSF1R blockade affects the differentiation of epithelial Paneth cells thereby affecting the adjacent intestinal epithelial stem cell populations, including their production of Wnt ligands (94). Moreover, blocking of CSF-1 signalling also impairs differentiation of other IEC lineages, including goblet cells and M-cells (94).

As implied by the above comments, the physiological crosstalk between IECs and myeloid cells in the gut is also closely regulated by the microbiota. Single cell RNA-sequencing (scRNA-seq) of colon myeloid cells from specific pathogen-free and germ-free mice showed that commensal microbiota specifically supported the generation of MФs but not DCs in the colon (95). In germ-free mice, the development of MФs is impaired, and their gene expression profile is affected with a downregulation of genes associated with immune defense and antigen presentation. These data highlight the importance of commensal microbiota on the unique developmental and functional diversification of colon MФs (95). Moreover, mouse strain with defective phagocyte activity revealed that insufficient bacterial elimination by mucosal myeloid cells can affect IECs differentiation and promote colon adenoma formation (96). More specifically, the persistence of bacteria within the LP potentiated the expression of the cyclooxygenase-2 enzyme by MФs, which in turn, induced epithelial tumor development (96, 97).

While traditionally much of the focus has been on directional signaling from myeloid cells to IECs, communication between these compartments is by no means unidirectional as myeloid cell differentiation is dictated not only by ontogeny (98) but also by environmental factors (99). Examples include the production of vitamin A-derived all-trans-retinoic acid (ATRA) by IECs in the small intestine, which controls the maintenance of CD103^+^ cDC2 in mice (100). Indeed recent studies suggest that ATRA, alongside mucus component, drives CD103^+^ cDC2 transcriptional and functional diversification into two distinct pools comprising a mature and proinflammatory phenotype, and a second intraepithelial cDC2 pool exhibiting immature and tolerogenic properties (101). Similarly, two subsets of cDC2 have been identified by transcriptome and epitope sequencing in human intestinal mucosa (102), although the biological functions of these two subsets in the human intestine remain unknown. Molecular factors derived from IECs in combination with other factors might also drive MФs diversification. Indeed, MФs from the distal colon are functionally, phenotypically, and transcriptionally different than MФs from the proximal colon (95), suggesting that IECs secrete different factors in the proximal colon *vs* distal colon (103) which could drive different MФ diversification.

### Crosstalk between innate lymphoid cells and intestinal epithelial cells

Epithelial cells detect bacteria/bacterial metabolites (danger signals PAMPs) *via* TLRs and translate it into co-stimulatory signals for ILC1s to produce IFN-γ (46). IFN-γ secretion by ILC1 is required for mucus secretion by goblet cells, which is important for protection of the barrier layer (48). This setup allows activation of ILC1s even in the absence of tissue injury. To avoid chronic activation of ILC1s, TGF-β was shown to dampen ILC1 activation and IFN-γ, but not TNF-α, secretion (104). In mice, several ILC2 effector cytokines can directly influence epithelial cells. Expression of the glycoprotein amphiregulin (Areg) by activated ILC2s promotes intestinal tissue protection (105) IL-25-induced release of IL-13 by ILC2s acts directly on intestinal progenitor cells and skews their differentiation towards tuft and goblet cells at the expense of absorptive enterocytes (53, 54).

IL-22 secreted by ILC3s directly acts on epithelial cells to promote repair and increased stem cell maintenance and proliferation in the murine gut. During bacterial infection, IL-22 maintains barrier integrity by directly promoting the proliferation and expansion of intestinal stem cells (106). The levels of IL-22 are regulated by DCs which secrete IL-22BP, myeloid cells which secrete IL-1β and IL-23 and epithelial cells which produce IL-1α. In addition, certain dietary components such as phytochemicals (glucosinolates) induce the expression of IL-22 through direct interaction with the transcriptional activator aryl hydrocarbon receptor (AHR) (107). IL-22/IL-22R signaling in epithelial cells induces the ATM serine/threonine kinase which is part of the DNA damage repair machinery protecting epithelial cells from genotoxic damage and part of the p53-dependent apoptosis pathway. AHR-mediated signaling in ILC3 promotes their differentiation and the establishment of cryptopatches and immune lymphoid follicles in the murine intestine. By contrast, excessive AHR signaling can lead to loss of ILC3, meanwhile a negative feedback loop involving AHR and cytochrome P450 leads to the reduction of dietary ligands of AHR, thus attenuating IL-22 expression and preventing loss of ILC3s (108). IL-22 also induces serum amyloid A secretion by murine intestinal epithelial cells which supports local T_H_17 responses and secretion of antimicrobial peptides to limit colonization with segmented filamentous bacteria (SFB) (109).

## Inflammation of the intestinal mucosa

### Myeloid cells in intestinal inflammation

Any perturbation in the stringent harmony that governs homeostasis leads to unwarranted immune activation inducing epithelial damage and inflammatory pathologies such as IBD, CD, and UC. IBD are chronic inflammatory diseases of the gastrointestinal tract of which immunopathology relates to an inappropriate and exacerbated mucosal immune response to components of the gut flora in genetically predisposed individuals (110). Intestinal MФs and DCs reside in the LP, and thus are ideally positioned to continuously sample intestinal luminal contents. On the other hand, intestinal barrier dysfunction precedes and predicts the development of CD (111, 112). In mice for instance, MФs in the distal colon sense water and fungal metabolite absorption by intestinal enterocytes and regulate the intestinal barrier in the colon through the aforementioned balloon-like protrusions. Depletion of those MФs leads to IEC death and loss of the intestinal barrier integrity which is a characteristic feature of IBD (21). By contrast the small intestinal barrier in mice is regulated by diurnal variations in food intake. Indeed, dietary timing and content has been shown to drive the microbiome composition and the transcriptional landscape of SI IECs; especially the expression of MHC-II and the production of IL-10 by epithelial cells and CD4^+^ T lymphocytes respectively. Disruption of this diurnal rhythmicity by alteration of the circadian clock results in enhanced microbial flux and exacerbation of Crohn’s-like enterocolitis (113).

Conventionally activated MФs play important roles in the pathogenesis of experimental colitis, through the production of inflammatory cytokines, reactive oxygen species (ROS) and nitric oxide (NO) (114). In particular, Th17 responses have been considered the main adaptive component of the pathogenesis of IBD, and Th17 responses are mediated by IL-1β, IL-6, IL-23 and TGF-β secreted by MФs (115). Primarily owing to their capacity to release IL-10, regulatory MФs are responsible for the resolution of colitis and the re-establishment of intestinal homeostasis (116). They work in concert with alternative activated M2 MФs that promote angiogenesis and debris scavenging, and support tissue repair of the disrupted epithelial barrier to prevent further unimpeded access of the luminal content to immune sentinels (117, 118). Accordingly, IL-10R-deficient patients with IBD, display defective functions of MФs as do patients with polymorphisms in the IL-10 promoter (119, 120), while mice deficient in IL-10 or IL-10R develop spontaneous colitis (121). Likewise, adoptive transfer of regulatory M2b MФs, activated by intravenous immunoglobulins or LPS, reduces intestinal inflammation in DSS-challenged mice and limit collagen deposition in the intestine (122) (**Figure 2B**). However, alternatively activated MФs may also contribute to fibrosis in CD, while regulatory MФs not only control colitis in mice, but also appear to play an active role in preventing the progression to fibrosis (122, 123).

Interestingly, some IBD susceptibility loci include regulatory regions of target genes for LPS or CSF-1-induced MФ differentiation (124). This suggests a link between defects in the resolution of intestinal inflammation and altered monocyte–macrophage differentiation that impairs bacterial clearance and results in excessive secretion of inflammatory cytokine in IBD patients (125, 126). Indeed, this process is dysregulated in both CD and UC, because both pathologies are associated with increased migration of CD14^High^ monocytes, leading to accumulation of CD11c^High^ inflammatory monocyte-like cells (127). Furthermore, colonic MФs in patients with CD show abnormal morphological maturation associated with prolonged survival of engulfed bacteria, resulting in excessive conversion and expansion of pathogenic T_H_17 cells (127).

Owing to the rapid turnover in the intestinal lining, timely phagocytosis of apoptotic intestinal epithelial cells and neutrophils is essential to prevent the excessive release of inflammatory cytokines by intestinal MФs during intestinal inflammation (25). Indeed, intestinal MФs with engulfed apoptotic intestinal epithelial cells overexpressed genes implicated in susceptibility to IBD (i.e., *IL12B*, *LSP1*, *SEPTIN1, IL12B*, etc), strongly suggesting that defective efferocytosis might contribute to the pathogenesis of IBD (25).

IBD pathogenesis is thought to be enhanced by improper MФ and DC responses to the microbiota (110). These responses include deficient protection and intensified pathogenicity. In a mouse model of T cell-induced colitis, inflammatory MФs accumulate in the intestine and the mesenteric lymph node where they produce inflammatory signals such as iNOS, and trigger the induction of pro-inflammatory T cells (128). Moreover, MФs isolated from patients with either CD or UC produce different cytokines in response to bacterial challenges. In CD, MФs produce more pro-inflammatory IL-23 and less anti-inflammatory IL-10, whereas in UC, MФs constitutively produce high levels of the pro-inflammatory cytokine IL-12. These increases in either IL-23 or IL-12 production, at the expense of IL-10 levels, contribute to the inflammatory exacerbation observed in UC and CD patients. Moreover, MФs from IBD patients show increased expression of TLR2 and 4, and therefore their increase susceptibility to bacterial products can exacerbate inflammatory responses and result in inflammation and autoimmune reactions (129).

Like MФs, LP-DCs promote tolerance to intestinal antigens at steady state but can become immunogenic upon inflammation or direct stimulation. Indeed, they can produce inflammatory cytokines, such as IL-12, IL-6, and IL-18, and thus induce T_H_1 responses when activated by TLR ligands (**Figure 2B**). Indeed, in IBD patients with active disease, circulating pDCs migrate to secondary lymphocytic organs resulting in the secretion of Th1 cytokines (IL-6, IL-8, TNF-α) thereby perpetuating disease (110). Meanwhile, inflamed and uninflamed ilea of CD patients harbor significantly fewer CD11c^+^ DCs, suggesting that loss of DC as may be a precursor to subsequent damage (130). However, despite the lesser abundance of CD103^+^ cDCs in IBD tissues, these cells have a potent ability to drive Th1/Th2/Th17 responses (131). More recent data on the contributions of individual cDC subsets remain controversial.

While some studies showed that cDC2 have no effect on DSS-induced colitis, others showed that cDC2s drive the initiation of T cell-driven colitis (132, 133). Indeed, deficiency of LP CD103^+^ cDC2 in human langerin-diphtheria toxin A (DTA) mice, or complete depletion of cDC2 in CLEC4a4-diphtheria toxin receptor (DTR) mice had no effect on the severity of colitis in response to DSS administration (132, 134). Nevertheless, impaired development of colon LP cDC2 in IRF4 conditionally depleted mice was associated with a delayed onset of T cell-dependent colitis (133) which suggested a role for IRF4-expressing cDC2 in the initial priming of colitogenic T cells.

Interestingly, cDC1 seem to have the capacity to protect/prevent colitis. cDC1 deficiency in XCR1-DTA mice, or depletion of cDC1 by administration of diphtheria toxin to CLEC9A-DTR mice, leads to enhanced susceptibility to DSS-induced colitis (34, 132).

### Innate lymphoid cells in intestinal inflammation

An imbalance in the composition of ILC subsets is commonly observed in preclinical models of IBD, CD and UC patients, and is thought to contribute to pathogenesis. The common theme emerging is the expansion of the pro-inflammatory natural cytotoxicity triggering receptor (NCR)^-^ T-bet^+^ ILC3 subtype at the expense of the tissue protective NCR^+^ T-bet^-^ ILC3 subtype accompanied by the increase in IFN-γ secreting ILC1 population while the frequency of ILC2 subtypes remains mostly unaffected (42, 43, 135–138) (**Figure 2B**).

IL-12, IL-18 and IL-1β produced by DCs in response exposure to microbial metabolites are the dominant cytokines required for the induction of IFN-γ and TNF-α released by ILC1s during gut inflammation (139, 140). IL-12 signaling (released by ILC1s) gives NCR^+^ ILC3 cells the ability to produce IFN-γ. Phenotypic conversion of NCR^+^ ILC3s to IFN-γ producing ILC1s is prominent in CD patients supporting the pathologic role for IFN-γ secreted by either ILC1s or ILC3s. Accordingly, mice lacking ILC1s were protected from experimental colitis (141). Of note, while both commensal and pathogenic gram- bacteria were able to induce IFN-γ in isolated gut ILC1s, gram^+^ bacteria failed to do so (142). A recent study has provided functional evidence showing activated ILC1 contributing to tissue remodeling in IBD through the secretion of TGF-β leading to epithelial growth matrix remodeling involving the protein kinase p38γ and the matrix metallopeptidase 9 (143). This raises the prospect that ILC1 may be able to facilitate tissue repair reminiscent to ILC2 and ILC3 subtypes *via* IL-13 and IL-22 secretion, respectively. Conversely, TGF-β was shown to antagonize the IL-15-mediated expression of cytotoxic molecules in an expanded population of IL-7Rα^+^ CD94^+^ ILC1s present in the lamina propria of patients with active CD but absent in healthy individuals (144).

Notwithstanding the well-established role of the IL-25-responsive, inflammatory subtype of ILC2s in tissue repair during intestinal parasite infections and their capacity to respond to microbial metabolites such as succinate, their contribution to IBD is less clear. Confounding results are mostly due to the role of the IL-33 cytokine, a major activator of ILC2 function. Expression of IL-33 in the epithelial cells is suppressed by the signaling protein Sprouty2 *via* the PI3K-Akt pathway and accordingly, loss of Sprouty2 in the colon protects mice from DSS-induced colitis. Notably, Sprouty2 is elevated in IBD patients suggesting an anti-inflammatory and tissue protective role for IL-33 (145). In addition to necrotic colonocytes, IL-33 is expressed from stromal cells, myeloid cells and endothelial cells in response to tissue damage and stress (146). This is most likely important since recent data in mice indicate that the cellular source of IL-33 dictates the biological consequences of IL-33 activity. While epithelial IL-33 drove host protective immunity, DCs-derived IL-33 suppressed it by controlling intestinal Treg numbers (147). A further source of IL-33 are microbiota-sensing mucosal macrophages. Here, the microbial product muramyl dipeptide, found in the walls of both gram^+^ and gram^-^ bacteria, is sensed by nucleotide-binding oligomerization domain–containing-2 (NOD2) expressing mononuclear phagocytes which in response secrete IL-33 which leads to IL-5 secretion in activated ILC2. Of note, NOD2 is a susceptibility gene associated with CD underscoring the relevance of this mechanism. IL-33 levels are reduced in NOD2 deficient mice, resulting in lower levels of ILC2 and acerbated inflammatory disease, indicative of a pathogenic role of the IL-33/ILC2 axis in the early stages of ileitis (148). In agreement with this, IL-33 abundance was elevated in colon biopsies of IBD patients (149) and IL-33 deficient mice were protected from DSS induced colitis while exogenous IL-33 aggravated disease. IL-33 deficiency resulted in impaired development of ILC2 and T_H_17 cells and reduced expression of pro-inflammatory cytokines IL-1β, IL-6 and IL-10 (150). In contrast to these findings, ST2 deficient mice displayed accelerated colitis in a DSS model and rIL-33 improved disease and induced strong upregulation of T_H_2 cytokines IL-5 and IL-13 as well as an accumulation of Tregs and ILC2s in the colon. Notably, murine Tregs were required for the induction for IL-5 and IL-13 and the expansion of ILC2 was independent of T and B cells (149, 151). However, it is currently unclear whether the expansion of Tregs in response to IL-33 requires the ILC2-M2 MФ axis as recently demonstrated in a murine lung sepsis model (151). Furthermore, thymic stromal lymphopoietin (TSLP) belongs to the family of ILC2-activating alarmin cytokines and is secreted mainly from epithelial cells and stromal cells. Its role in gut homeostasis and IBD is controversial. An initial older study reported increased TSLP expression in UC patients while two more recent studies report the opposite (152–154). IBD patients on anti-IL-13 treatment had increased TSLP expression and demontsrated improved tissue healing (153). TSLP receptor deficient mice did worse after DSS treatment and TSLP administration improved colitis by inducing TGF-β (155, 156).

During breaches of the epithelial barrier, bacterial metabolites trigger the release of pro-inflammatory cytokines IL-1β and IL-23 by tissue-resident macrophages which in turn activates ILC3s to release the cytoprotective IL-22. During colitis in mice, expression of IL-22 was negatively impacted by accumulating Tregs which suppressed the release of IL-23 and IL-1b from macrophages (78). In the intestinal tissue of UC and CD patients, the frequency of IL-17^+^ pro-inflammatory ILC3 subset (NKp44^-^) was increased and hyper-responsive to IL-23, while the tissue-protective NKp44^+^ ILC3 subset wass reduced (42, 136, 138, 157). Similarly, in a model of human neonatal necrotizing enterocolitis (NEC), the pro-inflammatory ILC3 subtype (NKp46^-^ RORγt^+^ T-bet^+^) was increased at the expense of the tissue protective ILC3 subtype (NKp46^+^ RORγt^+^) and ILC1 populations, while ILC2 frequencies were largely unaffected, despite a significant reduction in type 2 cytokines IL-4, IL-13 and reduced expression of Gata3. This suggests that T_H_2 cells rather than ILC2s were the source of the type 2 cytokines in this model (158).

There is limited data on pathways and mechanisms that suppress activation of ILC3 in inflammatory conditions such as IBD. Their identification is important for developing therapeutic approaches able to target ILC3 activity. Vitamin D downregulates IL-23 receptor signaling. Notably, vitamin D deficiency is a reported risk factor in IBD, hence vitamin D substitution may be beneficial by lowering IL-23 mediated induction of IL-17 secretion (159). The expression of circular RNA circKcnt2 which was induced in ILC3 during inflammation leads to suppression of IL-17 transcription and resolution of the inflammatory response. Mice lacking expression of this circular RNA exhibited activated ILC3s and aggravated colitis (160). Another avenue to curb ILC3 activity may be achieved through dietary means (161). Both high salt and high fat diets contributed towards disease progression (162, 163). A ketogenic diet (KD), characterized as high in fat and low in carbohydrates, was shown to alter the gut microbiota and microbial metabolites in mice, leading to ameliorations in both inflammation and disease severity after DSS. Mechanistically, levels of inflammatory cytokines (IL-22, IL-17 and IL-18), chemokines (CCL4, CCL12) and frequency of CP-resident ILC3s were reduced in mice on KD diet. This benefit was transferable through the fecal microbiota to mice on a normal diet. Microbial metabolites such as short chain fatty acids (SCFA) signal directly to mucosal ILC3 through binding to the GPR43 receptor (FFAR2) to induce ILC3 proliferation and release of IL-22 (164).

## Cancer in the intestinal mucosa

### Myeloid cells in colorectal cancer

IBD increases the risk of developing colorectal cancer (165). The emergence of single cell analyses has greatly facilitated the study of solid tumor microenvironments (TME) and their cellular diversity. scRNA-seq analyses on immune and stromal populations from colorectal cancer patients identified heterogeneity within the populations of tumor-associated macrophages (TAMs) and the tumor-associated DCs with diverse gene expression profile and functions. Comparing scRNA-seq from human colon cancer patients with mouse models of colon cancer, showed that the major tumor-associated myeloid cell populations are conserved in human and mouse (166). Moreover, computational modeling of all cell subsets enriched in tumors revealed a cell-cell interaction network in human and mouse colon cancer where TAMs and DCs harbor the most connections with other cell types (166). This predicted network suggests that TAMs and DCs subsets are central mediators of cellular crosstalk in the TME. In the next paragraphs we will briefly discuss MФ and DC populations present in colon cancer tissue.

Macrophages are one of the most important cells in the TME. They are recruited to the TME mainly by the chemokine CCL2, produced by cancer cells. Their role during tumorigenesis is complex because they have been associated with prevention and/or promotion of tumor development in different cancer types (167–170). Several studies have investigated the prognostic significance of MФ infiltration in colon cancer patients and have generally reported a correlation between better survival and high MФ infiltration (171–173), although conflicting reports exist (174, 175). As mentioned earlier, TAMs are traditionally segregated into pro-inflammatory and anti-inflammatory MФs, M1 and M2 respectively, but there is significant plasticity between these endotypes. For instance, suppression of an M2-like endotype is intracellularly controlled by distinct molecular mechanisms including kinase activities such as the gamma-isoform of PI3K (176, 177), or the hematopoietic cell kinase (HCK) (178) and others. Multiplexed immunofluorescence analyses using M1 and M2 specific markers revealed the presence of those TAMs in epithelial and stromal regions of human colorectal carcinoma (179). Tissue microarray analyses correlated with patient survival and revealed that, in tumor stroma, a high M1/M2 density ratio was associated with better cancer-specific survival (179). Similar observations have been made in human and mouse models of colorectal cancer (180–182). Indeed, preclinical models suggest that genetic or pharmacologic suppression of the M1 to M2 endotype transition reduces colon cancer in preclinical models (178) (**Figure 2C**).

It is now generally recognized that diverse phenotypic states exist between the two traditional M1-M2 polarizations. In colon cancer patients, two main populations of TAMs with distinct functional features have been identified by transcriptomic analyses (166): Secreted phosphoprotein 1 (SPP1) positive TAMs and complement C1q C chain (C1QC) positive TAMs. SPP1^+^ TAMs are enriched for pathways involved in tumor angiogenesis, tumor vasculature and colorectal adenoma, while C1QC^+^ TAMs are enriched for complement activation and antigen processing and presentation pathways. These observations suggest that SPP1^+^ TAMs played a pro-tumorigenic and pro-metastatic role in colon cancer whereas C1QC^+^ TAMs are involved in anti-tumor responses. Confirming these hypotheses, only C1QC^+^ TAMs are identified in the mucosal colon of UC and healthy individuals (183), whereas SPP1^+^ TAMs are virtually absent in non-cancer tissues (166). Notably, neither of these populations strictly correspond to the classically activated M1 or the alternatively activated M2 macrophages (**Figure 2C**).

TAMs have also been shown to participate in the development of colon cancer metastasis (184). They can secrete IL-6 and IL-11, two cytokines that engage the same signaling cascade associated with the shared gp130 receptor subunit which not only suppress apoptosis and enhance proliferation of neoplastic IECs, but also promote migration of colon cancer cells and induce the epithelial–mesenchymal transition program in cancer cells and therefore enhance metastasis (184, 185). Reciprocally, cancer cells produce CCL2 and GM-CSF which promote MФ recruitment (184, 186).

CSF-1 is a well-known regulator of MФ proliferation, differentiation, and survival. Clinical immunotherapies aiming at repressing TAM biology, by disrupting their expansion and differentiation, use anti-CSF1R which block CSF-1 binding to MФs. However, anti-CSF1R immunotherapy alone presents a low efficacy in patients with solid malignant tumors (187). *Zhang., et al.* showed, in mouse model of colon cancer, that anti-CSF1R treatment preferentially depleted a fraction of the C1QC^+^ TAM subset, while sparing SPP1^+^ TAM (166). Therefore, anti-CSF1R was insufficient to deplete MФ populations with tumor growth-promoting potential, hence its low clinical efficacy.

Conventional DCs (cDCs) have been widely studied in various immunogenic cancers, and researchers have shown that cDCs capture and transport tumor antigens to draining lymph nodes where they secrete pro-inflammatory cytokines such as IL-12, IL-6, TNF-α and IL-1β and (cross) present antigens to activate cytotoxic T lymphocytes [reviewed in (188)]. Similar to other malignancies, colon cancer antigens can induce DCs recruitment, maturation, and cytokine release in order to generate effective T_H_1-type immune responses (189). However, despite the key role of functional DCs in anti-tumor immunity, it is still largely unclear how colon cancer shapes DC fate. Indeed, immunosuppressive signals released by tumor cells or immunomodulatory cells, such as prostaglandin E2/cyclooxygenase-2, IL-10, TGF-β or vascular endothelial growth factor (VEGF), can induce DC dysfunctionality (190, 191). These molecules can modulate DC inflammatory responses by inhibiting their production of pro-inflammatory cytokines, and/or prevent DC maturation (**Figure 2C**).

Tumor-associated DCs, unlike TAMs, constitute only a minority of myeloid cells in the TME (166, 189). Little is known about how DCs are recruited to the TME in colon cancer, but a study in a mouse model of colon cancer showed that tumor-residing NK cells produce the chemokines XCL1 and CCL5 which attract cDC1 into the tumor where NK cells and other cells enhance their activation for efficient anti-tumor responses (192). Immuno-histological staining of human colon cancer tissue shows that the number of tumor infiltrating DCs is negatively correlated with survival, tumor size and metastasis (193–196). The level of DC maturation has been shown to be primordial for the correlation between the number of tumor-infiltrating DCs and patient prognosis. Indeed, only high infiltration with immature DCs (S100 positive) correlated with increased disease-free survival, while the presence of mature (HLA-DR^+^) DCs in the tumor epithelium showed an opposite effect on patient survival (193, 194, 196). In addition, a recent study on colon cancer patients indicated that the presence of DCs expressing the immunoinhibitory molecule PD-ligand 1 (L1) in the TME is associated with improved survival (197). Moreover, the density of PD-L1^+^ DCs in tumor compartments is positively correlated with the density of CD8^+^ cells, which suggests that the presence of PD-L1^+^ DCs reflects a hot immunological TME (197).

Contradictory observations have been made regarding pDC and survival prognosis in human colon cancer. While *Kiessler et al.* reported that a high tumor infiltration of pDC was correlated with prolonged survival of patients with colon cancer (198); Wu et al., showed that tumor-infiltrating pDC upregulated genes associated with tumor development and downregulated genes associated with tumor inhibition (199), suggesting that pDC participate in human colon tumor progression.

It is well established that immature DCs can induce tolerance. For instance, CD11b^+^ CD103^+^ cDC2 from the lamina propria were particularly competent at inducing Foxp3^+^ Tregs (200, 201). They do so through a TGF-β and RA-dependent mechanism: the small intestinal tissue constitutively expressed large amounts of TGF-β that triggered RA production by DCs which in turn converted CD4^+^ T lymphocytes into Foxp3^+^ Tregs (200, 201). Furthermore, it has been shown that the production of IL-27 by DCs, and especially cDC1, induced the development of type-1 regulatory T cells (202, 203) which enhanced tumorigenesis in mouse models of colon cancer.

### Innate lymphoid cells in colorectal cancer

Dysregulation in the composition, activation states and responses of immune cells in the TME of colon cancers are contributing events in the inception of pro- or anti-tumorigenic immune milieus (204). Recently, innate lymphoid cells (ILCs) have come to the fore as critical intermediaries during tissue homeostasis, inflammatory disease and cancer in the colon and are increasingly being recognized as potent and pleiotropic immunomodulators. In this section we will give an overview of recent developments, advances, and discoveries in this rapidly evolving field.

Until recently, no clear role for ILC1s in colorectal tumorigenesis had been described beyond their ability to promote chronic intestinal inflammation through their potent mediators IFN-γ and TNF-α. Nevertheless, both cytokines have well-documented anti-tumorigenic properties in both humans and mice: IFN-γ through stimulation of cytotoxic activity in T cells and NK cells and TNF-α through direct induction of apoptosis in tumor cells and tumor vasculature, and indirectly through mobilization of MФs and DCs into the TME (205–208) (**Figure 2C**). While NK cells fall outside the scope of this review, there have been few publications addressing the direct interplay between ILC1s and NK cells in colorectal cancer, as such, much of their functionality remains ambiguous and warrants further investigation (209). Studies directly addressing the role of ILC1 effector cytokines in colon cancer remain missing, instead the distribution and profiles of the various ILC subtypes in the periphery or inside the colon cancer tumor are being reported. A study identified a proliferating CD103^+^ intraepithelial ILC1-like population with cytotoxic activity localized in human colon cancers (210) while a CD56^+^ intraepithelial ILC1-like population was significantly expanded in the blood of metastatic colon cancer patients with the frequency of the corresponding ILC1 population decreased (211). More recently, single cell transcriptomics was used to profile ILCs in the blood and gut tissue of healthy individuals and colon cancer patients. Overall, the gene signatures were very similar between the ILCs isolated from healthy or diseased blood while the tumor ILCs were remarkably different. In particular, an ILC1-like subset was found to be colon cancer-specific. Notably, the authors identified the signaling lymphocytic activation molecule family member 1 (SLAMF1), a surface protein which was specifically and highly expressed in tumor ILCs (mainly ILC1-like and ILC2) and to a lower level in the blood of colon cancer patients but absent in the normal blood. Intriguingly, SLAMF1 is a potential predictive biomarker for colon cancer since the SLAMF1^high^ group of colon cancer patients had a better overall survival compared to the SLAMF1^low^ group (212). A similar observation was made in circulating ILC2s of patients with colitis (CD and UC) where SLAMF1 was upregulated on ILC2s in both patient groups compared to healthy control. Moreover, the frequency of *SLAMF1^+^* ILC2s in the blood was negatively correlated with disease severity in patients with active CD, but not UC (213). ILC1s expressed inhibitory receptors and underwent inhibitory functional conversion in late stage colon cancers while ILC1s in early-stage tumors expressed high levels of activating receptors (*KLRD1, NCR1, KLRC2, KLRB1C*) while late-stage colon cancers expressed inhibitory markers (*KLRE1, KLRA7*). Furthermore, late stage ILC1s were weak responders to IL-12, most likely due to loss of IL-12RB2 expression and reduced IFN-γ secretion (214).

ILC2s are abundant in colon cancer tissue and were the dominant source of IL-9. IL-9 was able to activate CD8^+^ T cells to inhibit tumor growth (**Figure 2C**). Conversely, blocking ILC2s promoted tumor growth in mice (215). Single-cell RNA profiling of ILC subsets in the AOM/DSS model of colon cancer identified 6 clusters of tumor infiltrating ILCs. ILC2s were classified into three subsets A, B and C with the ILC2-C subset shown to facilitate tumor progression. HS3ST1 (heparan sulphate3-O-sulfotransferase and programmed cell death protein 1 (PD-1) were highly expressed on ILC2s in late-stage tumors and lack of HS3ST1 and PD-1 in ILC2s suppressed tumor growth (214).

ILC3 numbers are reduced in colon cancer tissue compared to healthy controls in both humans and mice, mostly due to transdifferentiation of ILC3s into ILCregs during tumor progression. The conversion was partially mediated by TGF-β and inhibition of TGF-β signaling disrupted ILC3 conversion and curbed tumor growth (214). ILC3 to ILC1 transdifferentiation also resulted in increased inflammatory activity of T_H_17 cells, which is consistent with previous reports demonstrating an ILC3-dependent restriction of microbiota-specific T_H_17 activity during intestinal inflammation in an MHC-II-dependent manner (**Figure 2C**). Accordingly, deletion of MHC-II molecules on ILC3s accelerated tumor progression and aggressiveness and lead to resistance to anti-PD-1 checkpoint blockade while aslo eliciting changes to microbiota composition. This change in microbiota restricted the effectiveness of anti-tumor T_H_1 cell immunity and this defect was transferable by fecal transplants. IBD patients have reduced numbers of ILC3s and transfer of fecal microbiota transplant from IBD patients into mice reduced T_H_1 levels and increased resistance to PD-1 blockade (216). Numbers of ILC3s are negatively correlated with colon cancer stage, while plasmacytoid DC numbers are positively correlated. It was proposed that pDCs induced apoptosis of ILC3s through the Fas/Fas ligand pathway in the TME (199). A recent report highlighted the importance of microbial fungi in the regulation of colon cancer progression in mice (217). Here fungal control required pattern-recognition receptors such as dectin-3 expressed on MФs, and in dectin-3 null mice the increased fungal burden promoted colon cancer. Mechanistically, elevated fungal burden induced IL-7 production in dectin-3-deficient macrophages which then stimulated IL-22 release from ILC3s. In turn, IL-22 increased cancer cell proliferation in a Stat3-dependent manner. Notably, late-stage colon cancer patients had lower expressions of DECTIN-3 and conversely DECTIN-3 expression was high in patients with low fungal burden and high fungal burden predicted worse progression-free survival and overall survival. (218).

## Conclusion

A complex network of crosstalk between the epithelium and the innate and adaptive immune systems is required for the maintenance of intestinal homeostasis. While many of the mechanistic underpinnings of innate and adaptive immune cell functions have been deciphered through the utilization of mouse models, the functions of these cells in gastro-intestinal tract-associated diseases is continuously evolving.

As prolonged homeostatic imbalance can result in chronic inflammation, tumorigenesis and inefficient anti-tumor immune control, more scrutiny has been applied to the mucosal innate immune cells responsible for maintaining gut homeostasis. These tissue-resident immune cells are likely the first to sense changes in the gut environment, stemming from microbial dysbiosis *via* pattern recognition receptor on IEC (219) or *via* antigen presentation by DCs (220). Indeed, the close proximity of innate immune cells to the gut epithelium allows them to rapidly respond to invading pathogens, coordinating with the adaptive immune system to mount an effective defense. However, in many cases, aberrant activation of both innate and adaptive immune cells can promote chronic inflammation, an immunosuppressive environment, while increasing the risk of colorectal cancer development. Potentially due to a disturbance in the commensal microbial community, leading to gut dysbiosis and ongoing inflammation.

A deeper understanding of the contribution of individual immune cell subsets, and in particular of the myeloid and ILC lineages, are likely to unveil novel potential strategies to therapeutically manage IBD. Currently, clinical interventions for IBD are primarily based on the management of persisting inflammation through targeting of TNF-α, or the inhibition of IL-12 and/or IL-23 cytokines through p19 or p40-subunit specific antibodies, or the use of steroids to dampen the overall inflammatory response (221). In addition, anti-adhesion therapies with biologics targeting α4 and β7 integrin containing heterodimers or various sphingosine 1-phosphate receptors have shown promise in recent clinical trials (138, 222). While the inhibition of proinflammatory cytokines leads to a beneficial therapeutic effect predominantly by curbing the pathological activity of activated MФs, DC’s and ILC3s and an associated restoration of the ILC1/ILC3 balance, anti-adhesion therapies block the migration of leukocytes to the gut, their egress from the vasculature and the subsequent adhesion of intraepithelial T cells and DC’s to E-cadherin expressing epithelial cells (223). Therapeutic targeting of specific cellular subsets, either directly or indirectly through manipulation of the microbiome, is likely to allow a more patient-tailored approach and therefore potentially also reduce side-effects arising from current treatments. In mice for instance, eosinophil chemotaxis and associated spontaneous colitis is suppressed by treatment with a CCR3 receptor agonist (224), while the correction of dysbiosis in CD by fecal microbiota transplants is now being explored in Phase 2 clinical trial (NCT03078803).

Although there have been numerous attempts to target adaptive immune cells in colon cancer, both with anti-PD-1 and anti-CTLA-4 immunotherapies, this has been unsuccessful in >80% of colorectal cancers. In contrast, innate immunity in the intestinal epithelium can promote adaptive immunity, limiting cancer metastasis or recurrence. Most studies examining gut-resident innate immune cells (MAIT/γδ T cells) in colorectal cancer highlighted that the cytotoxic or activation phenotype of these cells is dependent on the expression of genes found in the tumor microenvironment or relies on *in vitro* stimulation. However, there is a lack of evidence and understanding if and how these cells become cytotoxicity deficient or anergic, resulting in reduced interaction with innate and adaptive tumor responding immune cells, impeding the killing of cancer cells. Indeed, preclinical studies using targeting innate immune cells in mouse models, suggest novel therapeutic strategies through inhibiting immunosuppressive MФs (178), activating ILC3s (216) and DCs (225), either alone or in combination with anti-PD-1/anti-CTLA-4 therapy in primary or metastatic colorectal cancer. Finally, the effect of targeting gut-resident ILCs and myeloid cells, through the identification of intestinal-specific regulatory or inhibitory immune markers could offer new therapeutic pathways for the treatment of CRC and need to be unraveled.

## Author contributions

SG, RO, LM, DR, MB and ME contributed to conception and design of the study. SG and RO made the illustrations. SG, DR and MB wrote the first draft of the manuscript. All authors contributed to manuscript revision, read, and approved the submitted version.

## Funding

This study was supported by the National Health and Medical Research Council of Australia (NHMRC) Program Grant (1092788) and Investigator Grant (1173814) and a Cancer Australia Grant (1144460) to ME, a NHMRC Project Grant (1143020) and a Tour de Cure VicDiscovery grant to MB, an Early Career Research Fellowship from the Victorian Cancer Agency (VCA) to DR and a VCA Mid-Career Research Fellowship and NHMRC Ideas grant (1185513) to LM and a La Trobe University Graduate Research Scholarship (LTGRS) to RO and the Operational Infrastructure Support Program, Victorian Government, Australia.

## Conflict of interest

The authors declare that the research was conducted in the absence of any commercial or financial relationships that could be construed as a potential conflict of interest.

## Publisher’s note

All claims expressed in this article are solely those of the authors and do not necessarily represent those of their affiliated organizations, or those of the publisher, the editors and the reviewers. Any product that may be evaluated in this article, or claim that may be made by its manufacturer, is not guaranteed or endorsed by the publisher.
